# Microscopic Evidence for the Topological Transition in Model Vitrimers

**DOI:** 10.1021/acsmacrolett.3c00586

**Published:** 2023-11-10

**Authors:** Arantxa Arbe, Angel Alegría, Juan Colmenero, Saibal Bhaumik, Konstantinos Ntetsikas, Nikos Hadjichristidis

**Affiliations:** † Centro de Física de Materiales (CFM) (CSIC−UPV/EHU) − Materials Physics Center (MPC), Paseo Manuel de Lardizabal 5, 20018 San Sebastián, Spain; ‡ Departamento de Polímeros y Materiales Avanzados: Física, Química y Tecnología (UPV/EHU), Apartado 1072, 20018 San Sebastián, Spain; ¶ Donostia International Physics Center (DIPC), Paseo Manuel de Lardizabal 4, 20018 San Sebastián, Spain; § Polymer Synthesis Laboratory, Chemistry Program, Physical Science and Engineering Division, KAUST Catalysis Center, King Abdullah University of Science and Technology (KAUST), Thuwal, 23955, Saudi Arabia

## Abstract

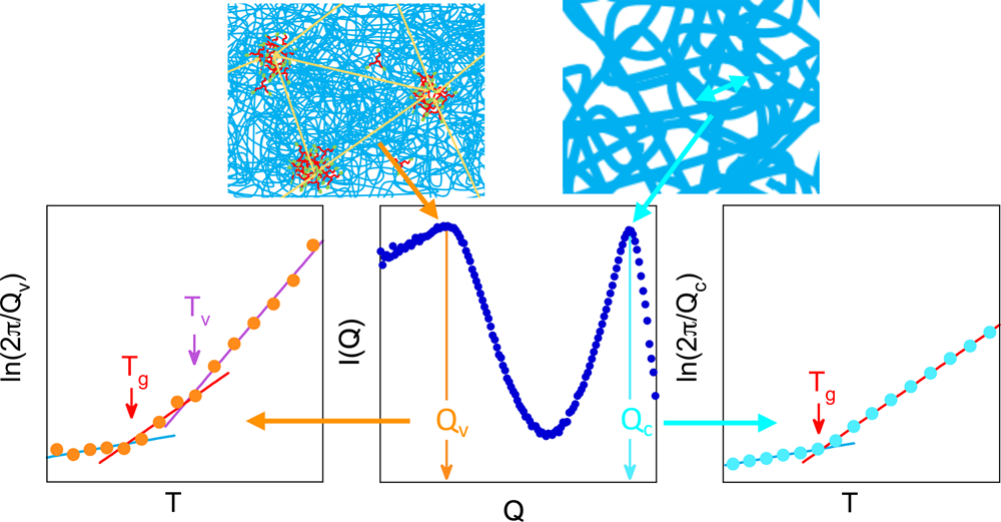

In addition to the glass transition, vitrimers undergo a topological transition from viscoelastic liquid to viscoelastic solid behavior when the network rearrangements facilitated by dynamic bond exchange reactions freeze. The microscopic observation of this transition is elusive. Model polyisoprene vitrimers based on imine dynamic covalent bonds were synthesized by reaction of α,ω-dialdehyde-functionalized polyisoprenes and a tris(2-aminoethyl)amine. In these dynamic networks nanophase separation of polymer and reactive groups leads to the emergence of a relevant length scale characteristic for the network structure. We exploited the scattering sensitivity to structural features at different length scales to determine how dynamical and topological arrests affect correlations at segmental and network levels. Chains expand obeying the same expansion coefficient throughout the entire viscoelastic region, i.e., both in the elastomeric regime and in the liquid regime. The onset of liquid-like behavior is only apparent at the mesoscale, where the scattering reveals the reorganization of the network triggered by bond exchange events. The such determined “microscopic” topological transition temperature is compared with the outcome of “conventional” methods, namely viscosimetry and differential scanning calorimetry. We show that using proper thermal (aging-like) protocols, this transition is also nicely revealed by the latter.

Since the publication of the seminal article by Leibler and co-workers^1^ the interest for vitrimers—the novel class of materials proposed in that work—has rapidly increased (see, e.g., the recent reviews^2−8^). Vitrimers consist of permanent networks containing dynamic bonds that allow the topology of the network to change, keeping always constant number of cross-links. Due to this property, in the viscoelastic regime, usually above the glass-transition *T*_g_, vitrimers can undergo a topological transition from a liquid to a solid (elastomeric) behavior. This transition is induced by the freezing, on the time-scale of observation, of the network rearrangements that are possible thanks to the dynamic bond exchange reactions. Thus, these systems combine the advantages of polymer thermosets—e.g., good mechanical properties, resistance to creep—and of thermoplastics—e.g., malleability, recyclability—being therefore excellent candidates for sustainable materials with good performance and versatility.^6^ In addition, their behavior is highly interesting from a fundamental viewpoint. Experimentally, following bond exchange reactions in bulk is not easy, and the observation of the topological transition is not trivial. The most common technique routinely used to determine transitions, differential scanning calorimetry, DSC, does not reveal clear features associated with this phenomenon. The most extensively applied methods to investigate vitrimers relate to their mechanical and rheological properties. Conventionally, the topological transition *T*_*v*_ is determined as the temperature at which the zero-shear viscosity reaches (by extrapolation) the value of 10^12^ Pa·s.^1,9^ This transition has also been monitored by dilatometry.^1,9,10^ Recently, AIE luminogens^11^ have been employed as a “non-invasive” probe for *T*_*v*_. To the best of our knowledge, a direct microscopic insight into this transition that would complement and support the macroscopic experiments without introducing additional elements is still missing.

On the other hand, nanophase separation has been reported for some supramolecular and covalent adaptable networks including vitrimer-like systems and vitrimers (see, e.g., refs (12−15)). Also, microphase separation is found in polymers with transient bonds, in systems that include immiscible chains and functional groups (see, e. g.^16,17^). This phenomenon introduces additional relevant length scales in the mesoscopic range related to domains characteristic for the cross-linking network. In this work, we have exploited this emerging feature to directly follow at the microscopic level the freezing of the topological changes of the network in polymeric vitrimers. The length-scale sensitivity offered by scattering techniques has allowed us to discern the impact of segmental mobility—associated with the universal α-relaxation of the polymeric phase—and bond exchange reactions—conferring the vitrimeric character to the system—on the structure factor of the material. The direct observation of the onset of the free volume contribution at the cross-linking points, triggering the viscoelastic liquid regime, has allowed the determination of the location of *T*_*v*_. The results agree well with those deduced from zero-shear viscosity measurements in the “conventional” way and DSC.

The samples investigated consist of vitrimers based on polyisoprene (PI) (see Figure 1). During the synthesis, the aldehyde groups at the ends of α,ω-PI chains react with the amino groups of the triamine [tris(2-aminoethyl)amine] cross-linkers, forming imine bonds. These are dynamic covalent bonds that confer the vitrimeric character to the system. The detailed synthesis and molecular characterization of precursors and final vitrimers is given elsewhere.^18^ The stoichiometry chosen is such that a small excess (≈ 5%) of primary amines is left, in order to facilitate bond exchange reactions.^19^ All chain ends are linked to a triamine since after the synthesis the samples were treated in solvent to wash out all unreacted component. Three lengths of the PI chains were probed (see Table 1). The concentration of triamine linkers increases with decreasing molecular weight *M* of the polymer, ranging from 0.8% for the highest *M* investigated (11k-vit sample) to 3.3% for the lowest (2k-vit sample). Two kinds of reactions involving the imine groups can take place: transimination and metathesis.^20^ The associated activation energies reported for these processes range between 10 and 130 kJ/mol.^6^

**Figure 1:**
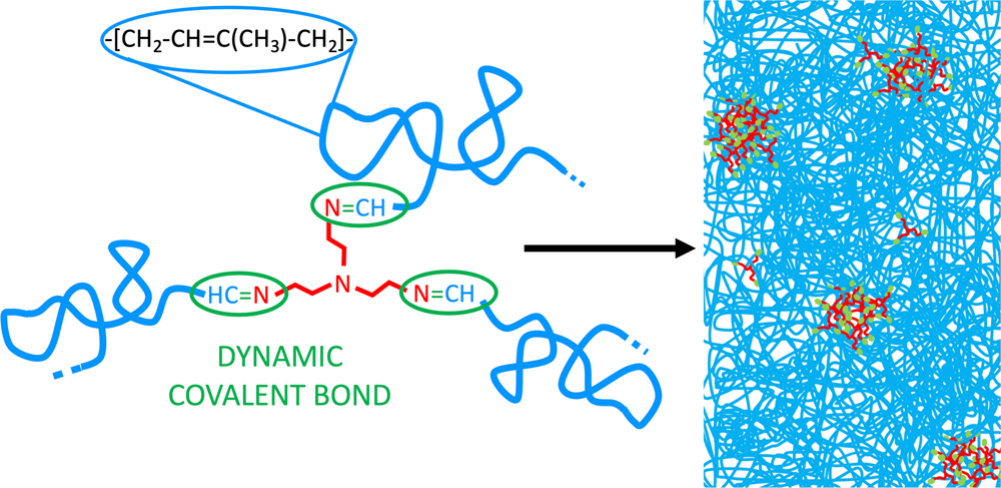
Schematic representation of the vitrimer showing the chemical composition. On the right, drawing of the nanosegregated structure suggested by SAXS experiments.

**Table 1:** Number-Average Molecular Weight (Determined by NMR), Polydispersity (Determined by SEC) for the Polymers, Volume Fraction of Triamine, Glass-Transition Temperature, Intercluster Average Distance at 300 K, Average End-to-End Distance for the Bulk PI Chains,^21^ and Expansion Coefficients in the Glass, Viscoelastic, and Liquid Regimes Determined from XR-Diffraction

Sample	*M*_*n*_ (kDa)	PDI	ϕ_*t*_	*T*_*g*_ (K)	*D* (Å)	⟨*R*_*e*,*o*_⟩ (Å)	β_*g*_ (K^–1^)	β_*v*_ (K^–1^)	β_*l*_ (K^–1^)
2k-vit	2.7	1.05	0.033	221.0	55	40	1.23 × 10^–4^	4.99 × 10^–4^	8.42 × 10^–4^
6k-vit	6.3	1.04	0.014	216.5	72	61	1.51 × 10^–4^	4.33 × 10^–4^	7.18 × 10^–4^
11k-vit	11.3	1.04	0.008	217.0	80	82	1.36 × 10^–4^	4.33 × 10^–4^	4.89 × 10^–4^

We first applied DSC with typical protocols (Supporting Information) to look for signatures of *T*_*v*_. Using standard heating/cooling rates of the order of 1 K/min, the heat capacity only shows a main feature, consisting of a clear step corresponding to the glass transition (see Figure S1 of the Supporting Information). From the inflection point, the values of *T*_*g*_ were obtained (see Table 1). They are clearly higher than those reported for standard PI samples with similar microstructure (about 65% 1,4-*cis*, 27% 1,4-*trans*, and 8% 3,4 units, in our case), that are 204–205 K,^22,23^ and also increase with respect to those obtained for the corresponding α,ω-hydroxyl functionalized PI homopolymers.^18^*T*_*g*_ is highest for the highest cross-linking density, as observed in regular networks.^24^ These observations can be considered as signatures of the presence of a cross-linking network, where the segmental mobility of the PI chains is restricted by the cross-linkers. At *T* > *T*_*g*_, no clear hint of an additional process can be found at first sight on the DSC results (see Figure S1). However, comparing the results with those obtained on “standard” PI, some very weak difference can be distinguished above ≈280 K (see the example of the 6k-vit sample in Figure S2 of the Supporting Information). The identification of this feature with a signature of the topological transition and a determination of the value of *T*_*v*_ from these results are, however, too speculative without additional support.

Parallel plate viscometry^25,26^ experiments that determine the zero-shear viscosity η_*o*_(*T*) were used to estimate *T*_*v*_ in the “conventional” way (*T*_*v*_^η_*o*_^). Degradation of samples limited the temperature range for the measurements. In the accessible range, the 11k-vit sample was too viscous to obtain conclusive results. The results obtained for the other two samples could be fitted with an Arrhenius law (see Figure S4 in the Supporting Information). The activation energies found are *E*_*a*_(2k-vit) = 66 ± 20 kJ/mol and *E*_*a*_(6k-vit) = 79 ± 7 kJ/mol. These values are in line with those found by dynamic rheology in ref.^18^ Assuming the extrapolation of this dependence up to η_*o*_ = 10^12^Pa·s, the value of *T*_*v*_^η_*o*_^ resulted in *T*_*v*_^η_*o*_^(2k-vit) = 272 ± 14 K and *T*_*v*_^η_*o*_^(6k-vit) = 274 ± 5 K. This estimated value of *T*_*v*_ would support attributing the weak calorimetric effect observed (see Figure S2) to the topological transition. However, we note that an Arrhenius extrapolation in the low-*T* range might not be appropriate,^27−30^ and the large uncertainty in the 2k-vit sample. A microscopic and definitive proof for this assignment in the three systems is thus needed. It is provided in the following, based on “microscopic dilatometry” experiments addressing the *T*-dependence of the structural features at different length scales.

The structure of the samples was investigated by XR diffraction. The results on the vitrimers at 300 K are shown in Figure 2, compared with those obtained on regular PI bulk polymers. In the high scattering vector (*Q*) range, we can see a well-defined and broad peak centered at *Q*_*c*_ ≈1.3 Å^–1^ independent of *M* and very similar to that present in standard PI. As for other linear polymers or polymers with small side groups, like e. g. 1,4-polybutadiene,^31^ this peak can be assigned to interchain correlations, i.e., correlations between pairs of atoms located at nearest neighboring chains.^32,33^ In the Bragg approximation, the average interchain distance *d* can be deduced from this peak position as *d* = 2π/*Q*_*c*_, being *d* ≈ 4.8 Å in all cases. On the contrary, moving toward larger length scales (lower *Q*-values), the emergence of a pronounced peak around *Q*_*v*_ ≈ 0.1 Å^–1^ evidences that the presence of linkers has a great impact in the structure. With increasing *M* of the polymer, the position *Q*_*v*_ shifts toward lower *Q*-values, indicating larger involved characteristic distances. The associated length scale *D* = 2π/*Q*_*v*_ ranges between *D* ≈ 55 Å (2k-vit) and 80 Å (11k-vit) (see Table 1). The origin of this low-*Q* peak must be attributed to correlations among linkers in the vitrimer. If they were homogeneously ditributed in the sample, the expected average distance between them would range between ≈19 Å (2k-vit) and ≈35 Å (11k-vit). These distances are much smaller than those deduced from the peak positions. This implies that the linkers have to be aggregated in clusters that are much more separated in space, being then *D* the value of the average intercluster distance in the systems. The broad feature of the peaks implies that there is a distribution of intercluster distances, and most probably, of cluster sizes. The estimated value for the average number of triamines in these clusters would be about 20, independently of *M* (see Supporting Information); accordingly, there would be of the order of 50 chain ends in the average cluster. Such a high concentration of imine groups should facilitate bond exchange reactions within the clusters. On the other hand, it is expected that PI chains connect different clusters. As a consequence, the average end-to-end distance ⟨*R*_*e*_⟩ of PI chains should be close to the average intercluster distance *D*. We can now estimate the values for ⟨*R*_*e*_⟩ expected in bulk for regular PI, ⟨*R*_*e*,*o*_⟩, using the relationship ⟨*R*_*e*,*o*_^2^⟩/*M* = 0.596 Å^2^ mol/g for PI at 298 K.^21^ These values are listed in Table 1. For the highest *M* investigated, *D* nearly coincides with ⟨*R*_*e*,*o*_⟩. This means that the conformation of the PI chains within 11k-vit is very close to Gaussian. As *M* decreases, *D* becomes somewhat larger than the Gaussian expectation. This suggests that the shorter chains would adopt slightly expanded conformations in the vitrimer. In any case, it follows that the conformational entropy of the polymer seems to play a crucial role in determining the resulting nanostructures. From the analysis of the XR results at 300 K, it can thus be concluded that the network shows a topology where cross-linking points are concentrated in regions connected by polymer chains in a nearly Gaussian conformation.

**Figure 2:**
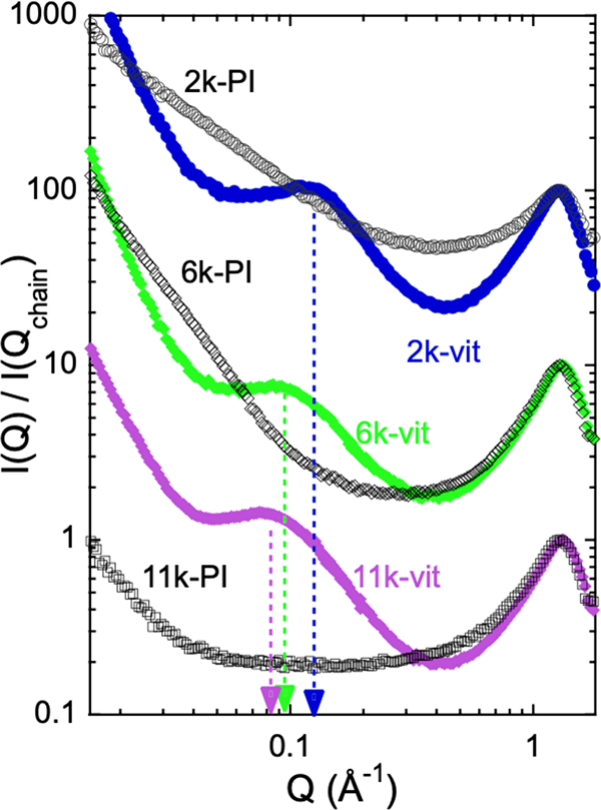
X-ray scattered intensity normalized to its value at the interchain peak for the three vitrimers investigated (solid symbols) at 300 K. Results obtained on regular PI samples of similar *M* for 2k-vit and 6k-vit and of 55 kg/mol for 11k-vit are shown for comparison (empty symbols). For clarity, data corresponding to intermediate molecular weight samples have been multiplied by 10 and those to low molecular weight by 100. Vertical arrows mark the positions of the intercluster peak.

Let us now consider the *T*-dependence of the structure factor at the different relevant length scales revealed by scattering experiments. Results for the 2k-vit sample are shown in Figure 3, and for the other systems in Figures S5 and S6 in the Supporting Information. The interchain correlation peak shifts toward lower *Q*-values with increasing *T* (panels a in these figures), reflecting the increase of the separation between PI chain segments as consequence of thermal expansion. For the three samples, the average interchain distance *d* shows a kink when plotted against *T*, that is located in the neighborhood of the calorimetric *T*_*g*_ (see Figure S7 in the Supporting Information). This kink reflects the different expansion in the glassy and supercooled liquid regimes due to the contribution of additional relaxation mechanisms, in particular, the segmental or α-relaxation—in the latter. The corresponding interchain expansion coefficients can be determined from the slope of ln[*d*(*T*)] (see Figure 4a–c), and have been included in Table 1 and Figure S8 in the Supporting Information. The values in the glassy state β_*g*_ are obviously lower than above *T*_*g*_ in the viscoelastic regime, β_*v*_. Importantly, we note that in this regime ln[*d*(*T*)] presents a fairly linear *T*-dependence, with no hint of an additional transition; i.e., the expansion of PI in the matrix only reveals the effect of the segmental dynamics. At the mesoscale, a *T*-dependence of the position of the maximum corresponding to the correlations between clusters is also observed (see panels b of Figure 3, S5 and S6). In Figure 4d–f, we show the *T*-dependence of the magnitude ln[*D*(*T*)]. To directly compare it with that at the interchain level, the slopes of the blue and red solid lines in these plots have been fixed to the values obtained for β_*g*_ and β_*v*_, respectively, from the interchain expansion results. Within the uncertainties, the thermal evolution of the intercluster correlations is perfectly accounted for by these expansion coefficients up to ≈260–270 K for the three samples. At higher *T*, a larger expansion is found for the clusters than that observed between polymer chains in the matrix where they are embedded. This extra expansion, as in a macroscopic dilatometry experiment, indicates the occurrence of an additional transition in the system; in this case, it should be the topological transition. Given the observable we are following, the transition must be related to an increase of the free volume available for the clusters, originating from additional degrees of freedom accessible thanks to the occurrence, within the time scale of observation, of imine bond exchanges. From these results we can thus determine the value of *T*_*v*_ as that above which topological changes contribute to expansion of the network. We chose the intervals marked by the shadowed areas in Figure 4 to account for the uncertainties. The values of the expansion coefficient in the viscoelastic liquid regime above *T*_*v*_, β_*l*_, are included in Table 1 (see also Figure S8 in the Supporting Information). They increase by increasing cross-linking density, as could be expected if imine bond exchange is behind this expansion. We could also speculate that in the systems with lower *M*, the network has a higher driving force to rearrange itself due to the observed stretched conformations of the polymers; in addition, shorter chains can diffuse more easily and lead to quicker rearrangements of the clusters. We note that in the 11k-vit sample the microscopic signature of the transition is subtle since β_*l*_ is not much higher than β_*v*_. In this system, the cross-linking density is the smallest, and overall chain mobility the slowest. Interestingly, concomitant with the change in the expansion, the *T*-dependence of the intensity of the peak also changes (see Figure 4d–f). Above *T*_*v*_, the peak becomes less intense than it would be expected from the evolution at lower *T*; in the case of the 11k-vit sample, it even decreases with *T* (actually, for this system, this magnitude provides a clearer fingerprint of the transition). This behavior might be due to two reasons: on the one hand, it can be related with fluctuations of the clusters around their equilibrium positions (thermal disorder), leading to a Debye–Waller factor modulation of the scattered intensity.^34,35^ This more disordered structure would be a consequence of reorganization of the clusters. On the other hand, it could reflect a loss of contrast between the polymeric matrix and the material within the clusters. This would be expected if the clusters are gradually dissolved and condensed again by migration of chains from one cluster to another and by motions of polymer segments within the clusters involved in bond exchange reactions that facilitate the reorganization of the network structure. Note that intercluster chain migration must occur in a concerted way, when a chain moves together with the other one(s) linked to the same triamine. Partial solubilization of domains could also be behind the observed evolution of both intensity and peak position.

**Figure 3:**
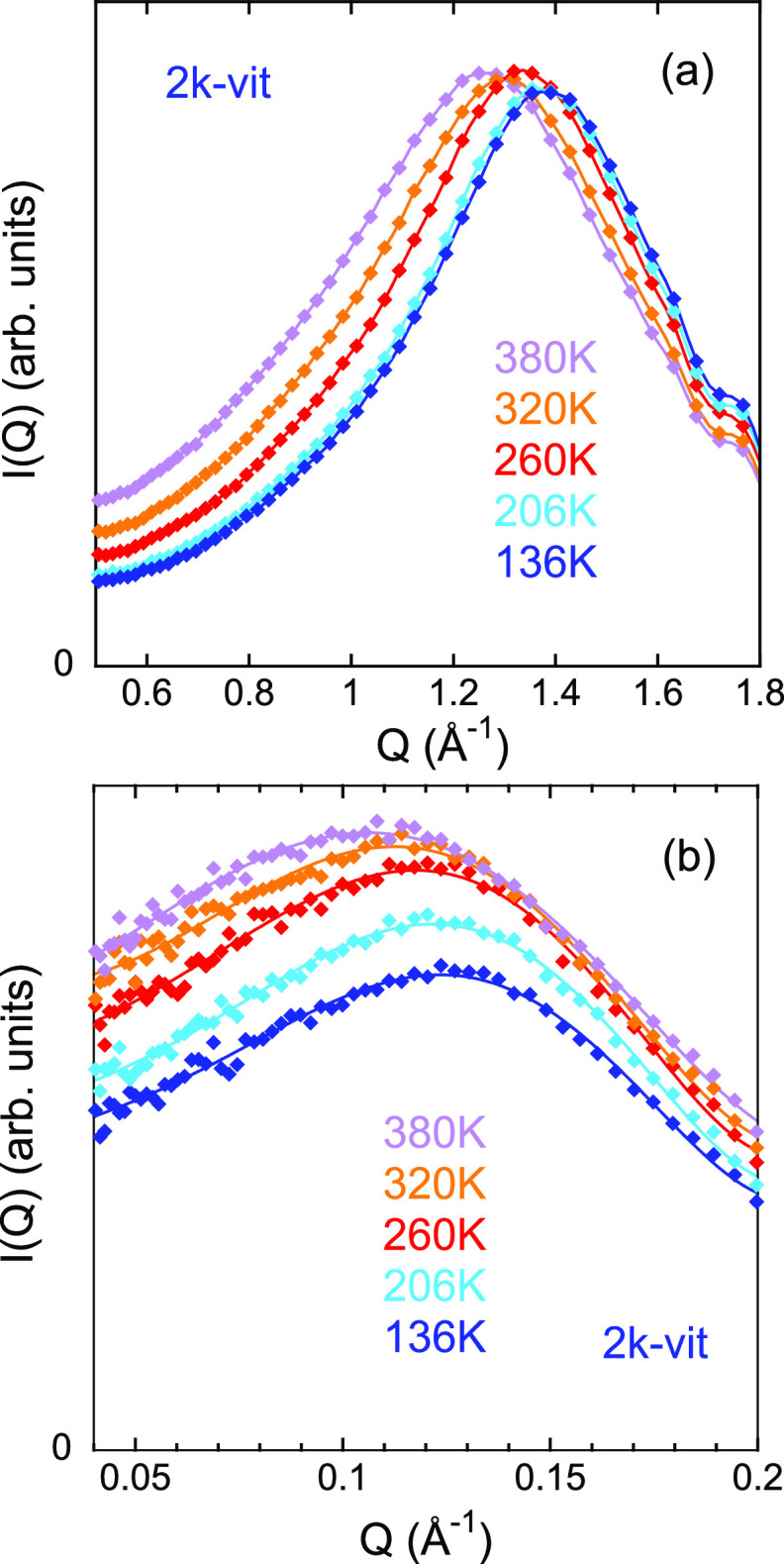
Temperature evolution of the interchain peak (a) and the intercluster peak (b) for the 2k-vit sample, at temperatures in the glassy state (136 and 206 K), close to the topological temperature (260 K) and above (320 and 380 K). In part b, the power-law component predominant at *Q* ≤ 0.04 Å^–1^ (see Figure 2) was subtracted from the intensity. Lines are guides for the eye.

**Figure 4:**
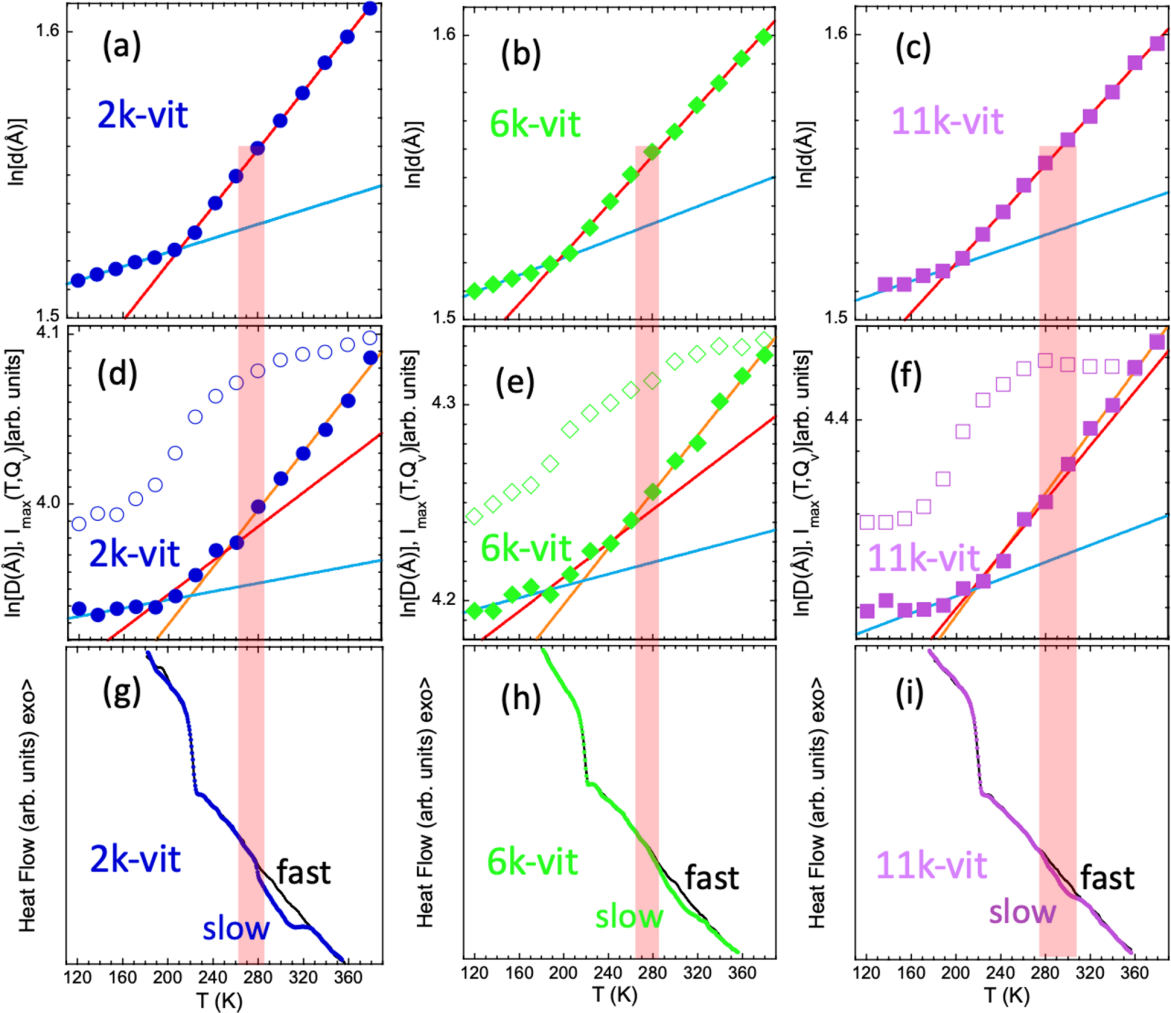
(a–c) Temperature dependence of the natural logarithm of the average interchain distance, ln(*d*). Lines are linear regression fits, yielding the expansion coefficients β_*g*_ and β_*v*_. (d–f) Temperature dependence of the natural logarithm of the average intercluster distance ln(*D*) (filled symbols) and of the intensity of the low-*Q* peak (empty symbols). The lines describing ln(*D*) in the neighborhood of the glass-transition have as slope the values of β_*g*_ and β_*v*_ deduced from the fits of ln(*d*). The orange lines in parts d–f are linear fits of ln(*D*) in the high-temperature regime yielding the expansion coefficient β_*l*_. (g–i) Calorimetric results obtained on heating at 20 K/min after fast cooling (≈70 K/min) (black lines) and after slow cooling (0.25 K/min) (points). Shadowed red areas indicate the locations of *T*_*v*_.

Comparing these microscopic results with the other techniques, the estimated onset of macroscopic flow agrees, within the uncertainties, with *T*_*v*_: *T*_*v*_^η_*o*_^ ≈ *T*_*v*_. The diffraction results have even allowed determining *T*_*v*_ for the 11k-vit sample where, as commented above, macroscopic viscosity is too high to use “conventional” methods. In addition, we notice that the weak feature detected by DSC could also be correlated with this microscopically determined *T*_*v*_ (see Figure S2). This observation motivated the design of a DSC protocol to enhance the calorimetric signature of the topological transition, inducing aging-like effects (see details in Supporting Information). The samples were subjected to a very slow cooling (0.25 K/min) from high *T* up to the glass transition, followed by a fast quench to 180 K. Thereafter, they were heated at 20 K/min, collecting data. The results are presented in Figure 4g–i and Figure S3 as points. They show a broad endothermic aging-like excess due to the difference in cooling–heating rates above the glass transition.^36^ This feature becomes more patent when compared with results obtained with very similar cooling/heating rates (black continuous lines in Figures 4g–i and S3). The area of the endothermic excess results to be proportional to the cross-linking density and is a signature of an underlying kinetic transition: the topological transition.

Summarizing, scattering sensitivity to structural features at different length scales has revealed how dynamical and topological arrests affect correlations at segmental and network levels. Chains expand obeying the same expansion coefficient—associated with degrees of freedom related to the segmental dynamics—throughout the entire viscoelastic region, i.e., both in the solid (elastomeric) regime and in the liquid regime. The onset of viscoelastic liquid-like behavior is only apparent at the mesoscale, where the scattering reveals features related to the reorganization of the network triggered by bond exchange events. The mesoscopic expansion exceeds the segmental one above *T*_*v*_, providing a clear fingerprint of the topological transition. The macroscopic results from zero-shear viscosity and DSC agree well with the location of *T*_*v*_ from these microscopic dilatometry experiments, which constitute a direct observation of the topological transition in model PI vitrimers.

## Supporting Information Available

Experimental procedures, estimation of domain size, and additional supporting figures regarding DSC, viscosity, and diffraction results. (PDF)
