# Supplementary material for: Microscopic Evidence for the Topological Transition in Model Vitrimers
Source: ACS Macro Lett. 2023 Nov 10;12(11):1595–601. doi: 10.1021/acsmacrolett.3c00586 (PMC10666534; doi:10.1021/acsmacrolett.3c00586)
Supplement: Supplementary file 1 — mz3c00586_si_001.pdf [file mz3c00586_si_001.pdf]

Supporting Information
Microscopic Evidence for the Topological Transition in Model Vitrimers

Arantxa Arbe,[*,†] Angel Alegría,[†,‡] Juan Colmenero,[†,‡,¶] Saibal Bhaumik,[§] Konstantinos Ntetsikas,[§] and Nikos Hadjichristidis[§]

†*Centro de Física de Materiales (CFM) (CSIC–UPV/EHU) – Materials Physics Center (MPC), Paseo Manuel de Lardizabal 5, 20018 San Sebastián, Spain*

‡*Departamento de Polímeros y Materiales Avanzados: Física, Química y Tecnología (UPV/EHU), Paseo Manuel de Lardizabal 3, 20018 San Sebastián, Spain*

¶*Donostia International Physics Center (DIPC), Paseo Manuel de Lardizabal 4, 20018 San Sebastián, Spain*

§*Polymer Synthesis Laboratory, Chemistry Program, Physical Science and Engineering Division, KAUST Catalysis Center, King Abdullah University of Science and Technology (KAUST), Thuwal, 23955 Saudi Arabia*

E-mail: a.arbe@csic.es

X-ray diffraction

Experiments were performed in a Rigaku 3-pinhole PSAXS-L equipment using CuK_α transition photons of $\lambda = 1.54$ Å. The 2D multiwire X-Ray Detector (Gabriel design, 2D-200X) is a gas-filled proportional type detector offering a 200 mm diameter active area with ca. 200 micron resolution. With three sample-detector distances the scattering vector (Q) range between 0.01 and 1.8 Å^{-1} can be covered. After azimuthal integration, the scattered intensities were obtained as a function of $Q = 4\pi \sin(\theta/2)/\lambda$, where θ is the scattering angle. Reciprocal space calibration was done using silver behenate and bromobenzoic acid as standards. Samples were placed in transmission geometry. Measurements under vacuum in isothermal conditions (1 h at each temperature) were performed by means of a Linkam Scientific Instruments THMS600 temperature controller with a temperature stability of ± 0.1 K. We explored 15 temperatures in the range 120 K$\leq T \leq$380 K. Stabilization times of 10 minutes were used before each measurement; experiments were performed on cooling, starting with the highest temperature investigated (380 K).

DSC

Differential Scanning Calorimetry (DSC) measurements were carried out on samples of approximately 10 mg placed in aluminum pans using a Q2000 TA instrument. A liquid Nitrogen Cooling System (LNCS) was used with 25 ml/min helium flow rate.

First, temperature-modulated experiments (MDSC) were performed using a sinusoidal variation of 1 K amplitude and 60 s period. Data were acquired during heating at 1 K/min from 180 to 350 K. The results for the three samples are presented in Figure S1. Figure S2 shows the results on the vitrimer system with intermediate molecular weight, 6k-vit, compared with those obtained on a reference PI sample with the same molecular weight. The red area shows the high-temperature region where some differences can be found between them.

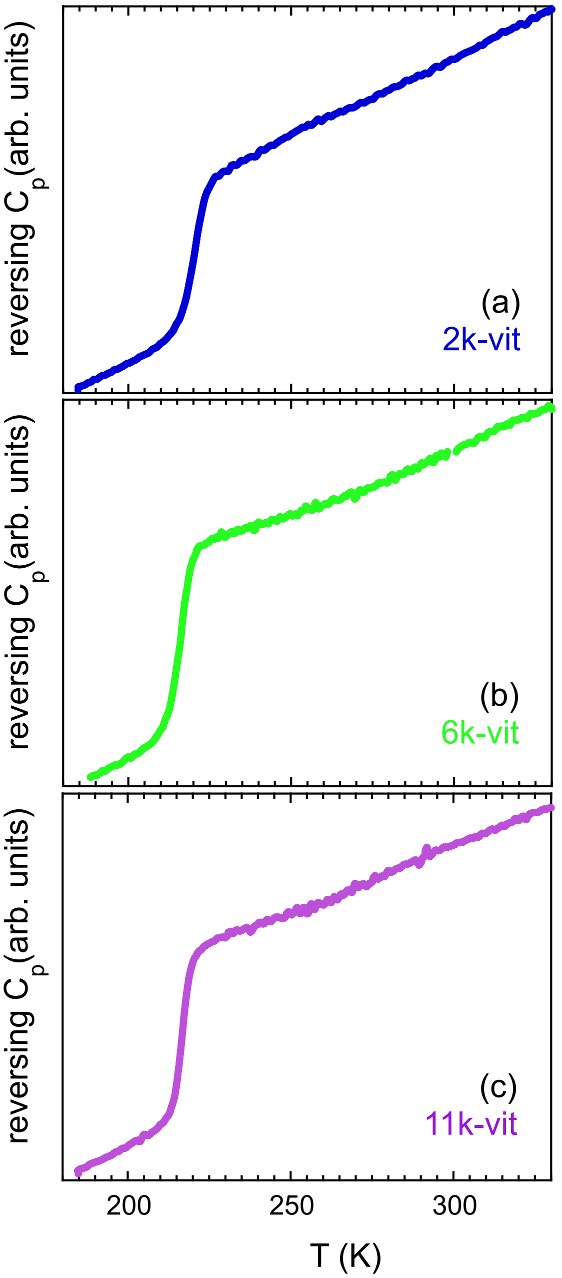

Figure S1: Reversible heat capacity obtained for the three vitrimers investigated with a rate of 1 K/min.

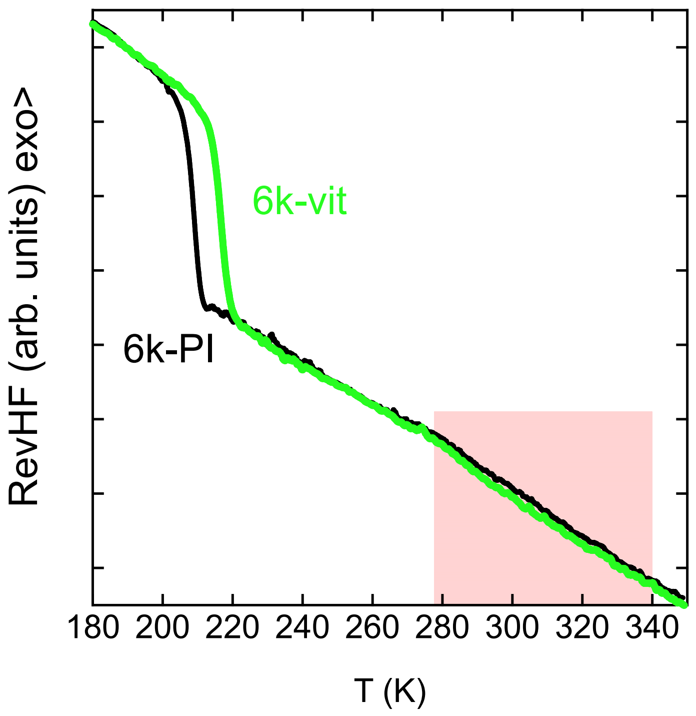

Figure S2: Reversible heat flow obtained for the 6k-vit sample (green curve) compared with that measured on a ‘standard’ PI-sample of the same molecular weight (black curve), both with a heating rate of 1 K/min.

A second type of experiments (aging-like) were conducted, in order to induce memory effects in the temperature region close to the vitrimeric transition. The idea behind was to cool down the system with a given (very slow) cooling rate, and record the results on heating with a very different (much higher) heating rate. These results would be compared with those obtained using the same heating rate, after applying a fast cooling (at a similar rate). Since we expected the vitrimeric transition to be located above the glass-transition, we applied the very different cooling/heating rates only above T_g. In practice, the sample was subjected to the following protocol to emphasize the effects not related to the glass transition process: (i) cooling with a rate of 0.25 K/min, down to 220 K (i. e., just above the glass transition temperature); (ii) cooling down to 180 K with the highest possible rate (which, in this temperature range, resulted to be about 30 K/min); (iii) heating at 20 K/min up to 360 K; (iv) cooling down to 180 K with the highest possible rate (which around RT is about 70 K/min); (v) heating at 20 K/min up to 360 K. Last, steps (iv)-(v) were repeated to check reproducibility. With this sequence, in steps (i) and (iii) we applied very different heating/cooling rates (ratio: 80) where we expected T_v to occur, while in the region of T_g (steps (ii) and (iii)) the rates were rather similar (approximate ratio: 1.5). On the other hand, the results of steps (iv) and (v) can be considered as the reference where similar heating and cooling rates are applied (approximate ratio: 0.3).

Figure S3 shows for the three vitrimers investigated the results obtained during steps (iii) and (v), in the temperature range above the glass-transition temperature.

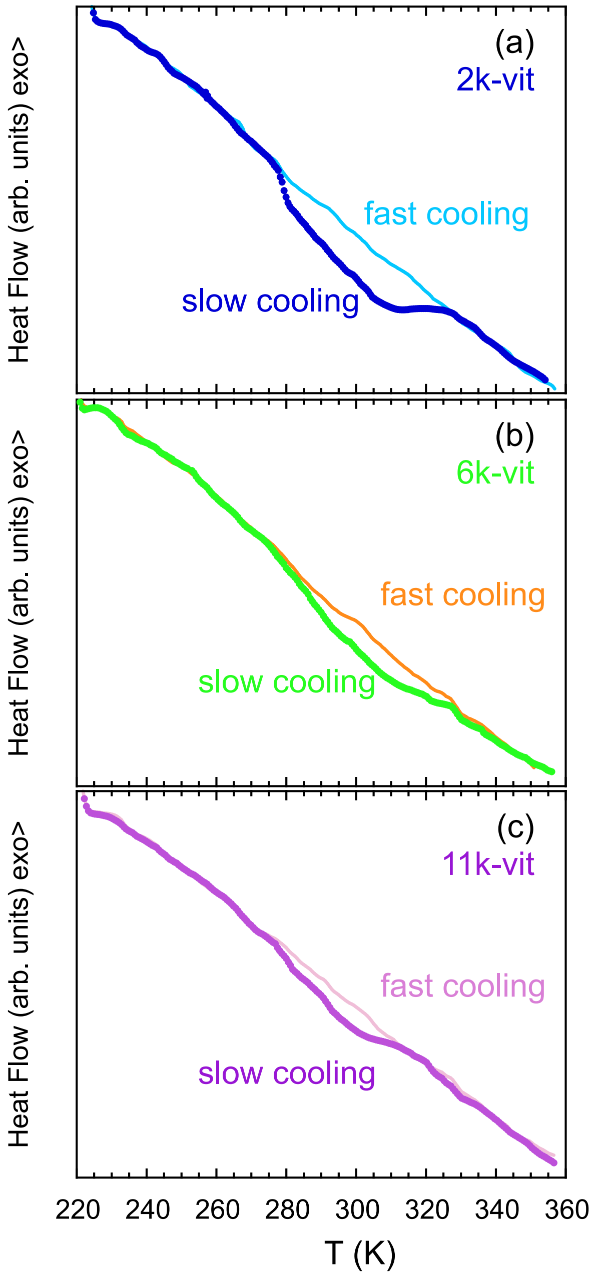

Figure S3: Heat flow measured on the three vitrimers during step (iii), i. e., after a ‘slow’ cooling at 0.25 K/min in the viscoelastic region (points), and during step (v), i. e., after a fast cooling of about 70 K/min (lines).

Parallel plate viscometry

Parallel plate viscosimetry experiments[1,2] were performed using a LINSEIS Zero Friction L75 apparatus by recording the time evolution of the sample thickness. Sample discs of 4 mm diameter and about 0.5mm thickness were subjected to a constant compressing force of 1 N. Typical experimental time at each temperature was a few hours and the values reported at each temperature were obtained from four different experiments. The results are displayed in Fig. S4.

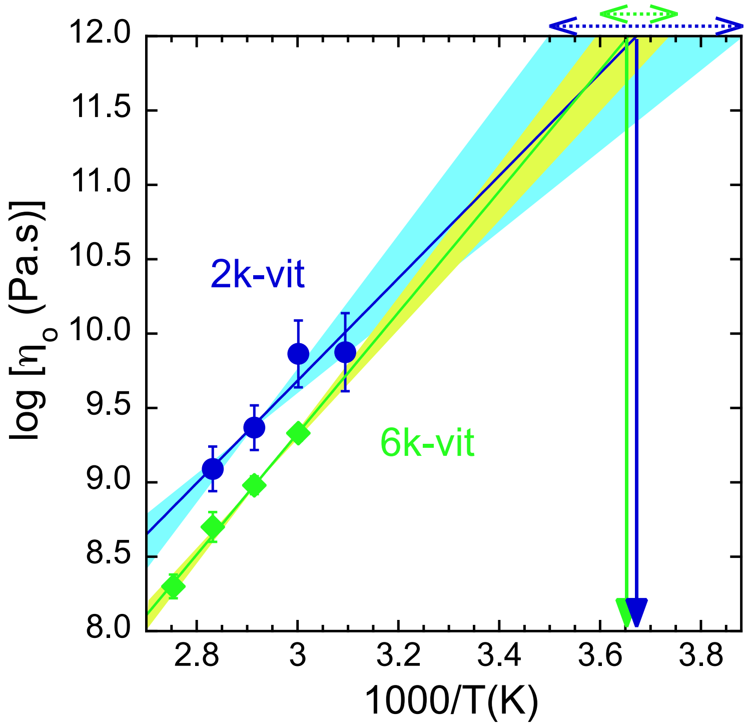

Figure S4: Inverse temperature dependence of the zero-shear viscosity obtained for the 2k-vit sample (circles) and the 6k-vit sample (diamonds). Lines are Arrhenius fits. Vertical arrows mark the inverse of the topological temperature $T_v^{\eta_o}$ from the extrapolation to the value $\eta_o = 10^{12}$Pa·s. Shadowed areas represent the uncertainty associated to the determination of the activation energy, translated in the uncertainty in $T_v^{\eta_o}$ indicated by the horizontal arrows.

Estimation of domain size

Applying a very simple model –a cubic arrangement of spheres of triamines with radius r_t with D the side of the cell– we can estimate the size of the cluster as $r_t = D[3\Phi_t/(4\pi)]^{(1/3)}$.[3] The resulting values of r_t are of about 10–11Å in all cases. This implies that, in this simple scenario, each cluster would contain about 20 triamine molecules. Taking into account that each triamine is linked with up to three PI chains and the excess of amino groups, we would expect of the order of 50 chain ends per cluster. Obviously this model is oversimplified. First, the cluster cannot contain exclusively triamines, but also some adjacent chain segments of the most inner triamines. Thus, the radius of the cluster should be larger than 10–11Å, considering the embedded polymer segments. Second, it has been assumed that all triamines belong to the clusters.

Temperature dependence of the peaks

Figures S5 and S6 show representative X-ray diffraction results for the 6k-vit and 11k-vit samples, respectively, at different temperatures. The temperature dependence of the interchain distance d obtained for the three vitrimers is shown in Fig. S7. Figure S8 displays the molecular weight dependence of the expansion coefficients.

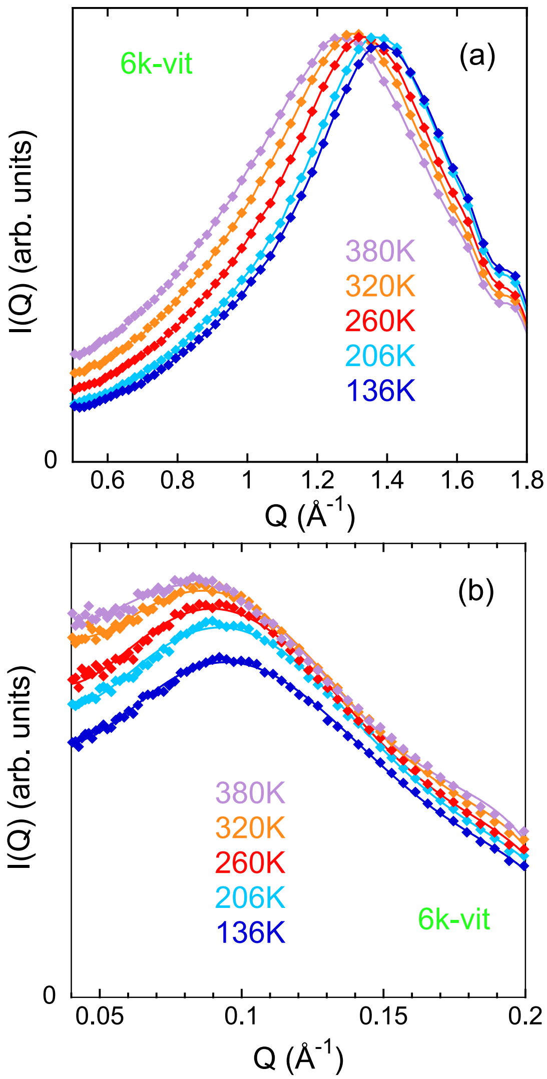

Figure S5: Temperature evolution of the inter-chain peak (a) and the inter-cluster peak (b) for the 6k-vit sample, at temperatures in the glassy state (136 and 206 K), close to the topological temperature (260 K) and above (320 and 380 K). In (b), the power-law component predominant at $Q \leq 0.04\text{Å}^{-1}$ (see Fig. 2) has been subtracted from the intensity. Lines are guides for the eye.

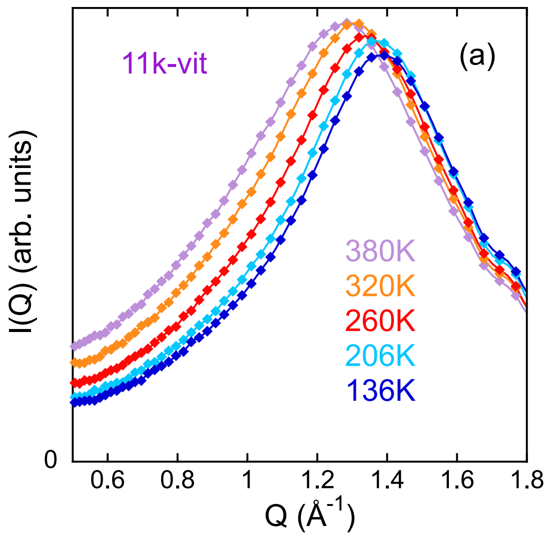

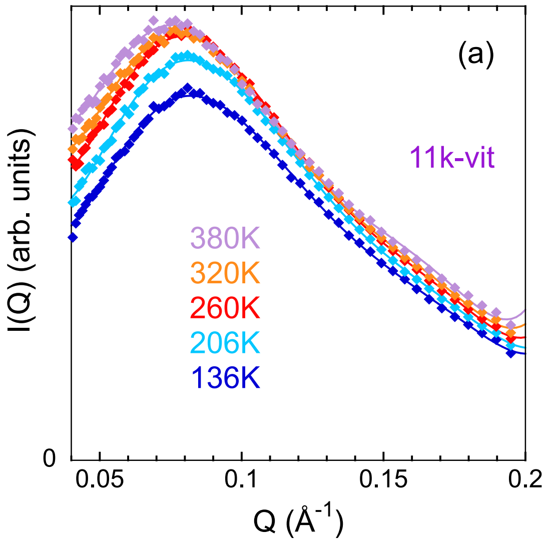

Figure S6: Temperature evolution of the inter-chain peak (a) and the inter-cluster peak (b) for the 11k-vit sample, at temperatures in the glassy state (136 and 206 K), close to the topological temperature (260 K) and above (320 and 380 K). In (b), the power-law component predominant at $Q \leq 0.04$Å^{-1} (see Fig. 2) has been subtracted from the intensity. Lines are guides for the eye.

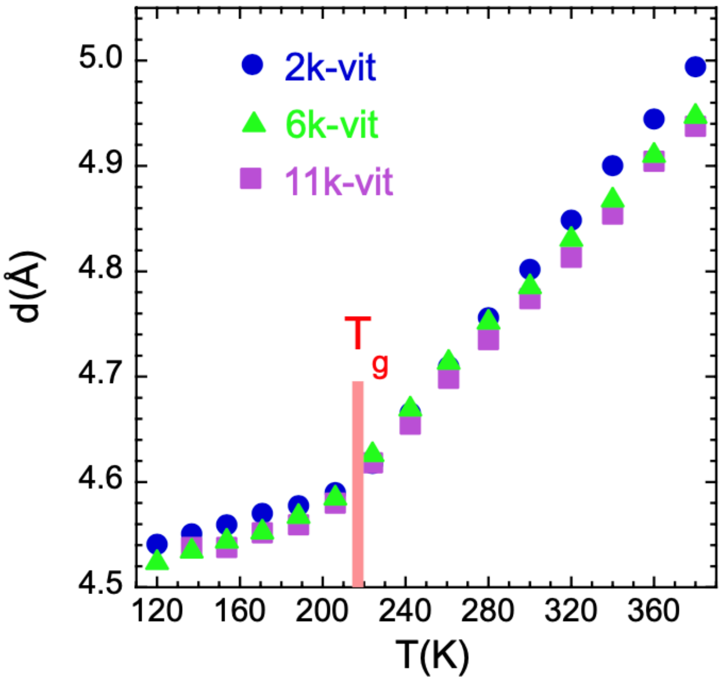

Figure S7: Temperature dependence of the average inter-chain distance for the three vitrimers investigated. The red shadowed area marks the region of the calorimetric glass-transition temperatures.

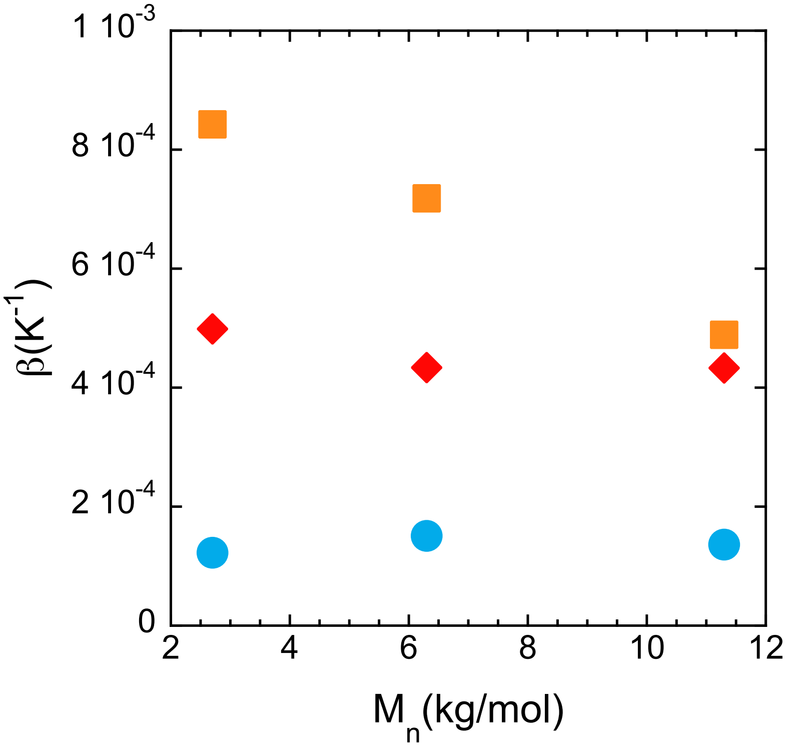

Figure S8: Molecular mass dependence of the expansion coefficients in the glassy state (β_g, blue dots) and in the viscoelastic regime (β_v, red diamonds) determined from the inter-chain peak, and in the viscoelastic liquid regime (β_l, orange squares) from the inter-cluster peak.

References

(1) Gent, A. N. Theory of the parallel plate viscometer. *British Journal of Applied Physics* **1960**, *11*, 85, DOI: 10.1088/0508-3443/11/2/310.

(2) Macho, E.; Alegría, A.; Colmenero, J. Determining Viscosity Temperature Behavior of Four Amorphous Thermoplastics Using a Parallel Plate Technique. *Polymer Engineering and Science* **1987**, *27*, 810–815, DOI: 10.1002/pen.760271107.

(3) Ge, S.; Samanta, S.; Tress, M.; Li, B.; Xing, K.; Dieudonn-George, P.; Genix, A.-C.; Cao, P.-F.; Dadmun, M.; Sokolov, A. P. Critical Role of the Interfacial Layer in Associating Polymers with Microphase Separation. *Macromolecules* **2021**, *54*, 4246–4256, DOI: 10.1021/acs.macromol.1c00275.
